# A Deeper Look at Autonomous Vehicle Ethics: An Integrative Ethical Decision-Making Framework to Explain Moral Pluralism

**DOI:** 10.3389/frobt.2021.632394

**Published:** 2021-05-04

**Authors:** Jimin Rhim, Ji-Hyun Lee, Mo Chen, Angelica Lim

**Affiliations:** ^1^ Robots with Social Intelligence and Empathy (ROSIE) Lab, School of Computing Science, Simon Fraser University, Burnaby, BC, Canada; ^2^ Multi-Agent Robotic Systems (MARS) Lab, School of Computing Science, Simon Fraser University, Burnaby, BC, Canada; ^3^ Information-based Design Research Group, Korea Advanced Institute of Science and Technology (KAIST), Graduate School of Culture Technology, Daejeon, South Korea

**Keywords:** autonomous vehicle, AI ethics, ethical decision-making, moral pluralism, explainability, transparency

## Abstract

The autonomous vehicle (AV) is one of the first commercialized AI-embedded robots to make autonomous decisions. Despite technological advancements, unavoidable AV accidents that result in life-and-death consequences cannot be completely eliminated. The emerging social concern of how an AV should make ethical decisions during unavoidable accidents is referred to as the moral dilemma of AV, which has promoted heated discussions among various stakeholders. However, there are research gaps in explainable AV ethical decision-making processes that predict how AVs’ moral behaviors are made that are acceptable from the AV users’ perspectives. This study addresses the key question: What factors affect ethical behavioral intentions in the AV moral dilemma? To answer this question, this study draws theories from multidisciplinary research fields to propose the “Integrative ethical decision-making framework for the AV moral dilemma.” The framework includes four interdependent ethical decision-making stages: AV moral dilemma issue framing, intuitive moral reasoning, rational moral reasoning, and ethical behavioral intention making. Further, the framework includes variables (e.g., perceived moral intensity, individual factors, and personal moral philosophies) that influence the ethical decision-making process. For instance, the framework explains that AV users from Eastern cultures will tend to endorse a situationist ethics position (high idealism and high relativism), which views that ethical decisions are relative to context, compared to AV users from Western cultures. This proposition is derived from the link between individual factors and personal moral philosophy. Moreover, the framework proposes a dual-process theory, which explains that both intuitive and rational moral reasoning are integral processes of ethical decision-making during the AV moral dilemma. Further, this framework describes that ethical behavioral intentions that lead to decisions in the AV moral dilemma are not fixed, but are based on how an individual perceives the seriousness of the situation, which is shaped by their personal moral philosophy. This framework provides a step-by-step explanation of how pluralistic ethical decision-making occurs, reducing the abstractness of AV moral reasoning processes.

## Introduction

With recent artificial intelligence (AI) advancements, robots are expanding from conducting predefined tasks in confined environments to becoming autonomous agents in real-world contexts. Autonomous vehicles (AVs) are among the most significant commercialized AI-embedded autonomous agents that reflect this technological transition. A report of Americans’ long-term adoption of AVs forecasts mass production of AVs with high automation by 2024 (Bansal and Kockelman, 2017). The adoption of AV promises many benefits that improve transportation experiences such as reduced costs, more rest time for vehicle users, mobility to nondrivers, and minimized pollutions (Schoettle and Sivak, 2014; Fagnant and Kockelman, 2015). Most importantly, AVs are expected to increase road safety by reducing the number of accidents and severity of crash consequences by making more rational decisions (Anderson et al., 2014; Kumfer and Burgess, 2015; Nyholm and Smids, 2016; Gogoll and Müller, 2017; Hulse et al., 2018).

Despite these technological advancements, AV accidents cannot be entirely eliminated (Goodall, 2014b; Bonnefon et al., 2016; Guo et al., 2018; Nyholm and Smids, 2018). In this regard, AVs are among the first autonomous agents that make decisions with potential life-and-death consequences (Awad et al., 2020). While vehicle accidents have existed, the introduction of AVs has shifted ethical implications during accidents Danielson (2015), Shariff et al. (2017), Awad et al. (2018a), Taddeo & Floridi (2018) because humans and AVs make intrinsically different ethical decisions. In conventional accidents, human drivers tend to show crash avoidance behaviors Lerner (1993), Yan et al. (2008) within 2 s of reaction time Lin (2015), resulting in reflexive and instinctive decisions (Goodall, 2014a). Thus, human decisions or driving behaviors cannot be held morally accountable (Goodall, 2014b; Lin, 2015; Shariff et al., 2017). In contrast, AVs are equipped with advanced sensors and preprogrammed algorithms that can anticipate and react to accidents better than human drivers. Therefore, AV decisions that impact human lives are preprogrammed (Goodall, 2014a; Carsten et al., 2015; Karnouskos, 2020b). The decision of an AV to protect whom or what during an emergency falls into distributing harm, a universally agreed-upon moral domain (Haidt, 2001). As an AV is an artificial moral agent capable of making decisions with ethical consequences (Allen et al., 2005; Wallach et al., 2010), an in-depth understanding of AV ethics is necessary.

The emerging social concern of how AVs should behave ethically in unavoidable crashes started a heated discussion in AV ethics, which is referred to as the moral dilemma of AVs (Bonnefon et al., 2016; Gogoll and Müller, 2017; Goodall, 2014a, Goodall, 2014b; J. Greene, 2016; Hevelke and Nida-Rümelin, 2015; Lin, 2015; Nyholm and Smids, 2016). The most dominantly discussed AV ethical issue is based on an extension of the trolley problem Goodall (2014a), J. Greene (2016), Lin (2015), Shariff et al. (2017), which asks whether people prefer deontology (determining good or bad based on a set of rules) or utilitarianism (determining good or bad based on outcomes) (Gawronski and Beer, 2017). However, many researchers are dismissive of AV ethics based on the trolley problem for the following reasons. First, the hypothetical scenarios adopted in the thought experiment are too simplified and ambiguous (Gawronski and Beer, 2017; De Freitas et al., 2020b). In fact, most scenarios in AV moral dilemmas tend to focus mainly on the consequences made from predefined binary choices, e.g., the number or characteristics of people who are impacted. This approach disregards other important AV crash-related factors such as regulations, responsibilities, or moral norms. Second, the results are highly likely to be biased. Trolley problem-based scenarios often begin by favoring a specific moral theory, resulting in a biased interpretation of the results (Dubljević and Racine, 2014). Studies have shown a discrepancy between people’s preference and acceptance of utilitarian AVs due to this bias. For instance, people answered that they prefer utilitarian AVs that save more lives but would not purchase such AVs, as they might sacrifice themselves (Bonnefon et al., 2016; Shariff et al., 2017; Awad et al., 2018a). Third, ethical decisions based on the trolley problem tend to be unfair (Goodall, 2014a; J. Greene, 2016; Taddeo and Floridi, 2018). The results reveal people’s preferences to determine who to kill based on personal features (e.g., save women and kill men) Bigman & Gray (2020), which disregards the equal right to human, an integral ethical concern (Kochupillai et al., 2020). Further, such unfair preferences violate the Rule 9 of German Ethics Code for Automated and Connected Driving, which strictly prohibits discrimination based on personal features (Luetge, 2017). As a result, people are angered at AVs that make prejudiced decisions (De Freitas et al., 2021). Consequently, public fear and outrage could delay the adoption of AVs (Shariff et al., 2017). Finally, trolley problem-based AV ethics tends to rely on a single moral doctrine (e.g., utilitarian). Relying only on one specific moral principle cannot explain complex real-world values. Indeed, human morality is pluralistic (Graham et al., 2013; Schoettle and Sivak, 2014; Fagnant and Kockelman, 2015). Therefore, providing AV ethical perspectives other than utilitarianism needs to be considered (Dubljević, 2020). To overcome the limitations of the trolley problem-based AV ethics, an alternative approach that incorporates varying human values and crash contexts should be considered.

Providing explainable AV moral behaviors is essential to ensuring the transparency of AV systems (J. Greene, 2016). One way to achieve this goal is to develop an AV framework that explains and predicts the full ethical decision-making process Winfield et al. (2019), Karnouskos (2020a) matching end-users’ values (Bonnemains et al., 2018). AV ethics requires a collaborative and interdisciplinary effort from technical, regulatory, and social spheres (Borenstein et al., 2019; De Freitas et al., 2020a; De Freitas et al., 2020b; Mordue et al., 2020). Therefore, it is integral for various stakeholders (e.g., AV developers, engineers, regulators, ethicists, and social scientists) to have an open discussion about forming value-aligned moral behaviors of AV Goodall (2014b), De Freitas et al. (2020b). As AI-based reasoning is a blackbox Castelvecchi (2016), AV moral reasoning will be challenging to fully understand, even for those who programmed them. Furthermore, AVs are mostly elaborated by engineers, transportation experts, policy makers Bansal and Kockelman, (2017) and AI ethicists Vrščaj et al. (2020) lacking prospective AV users’ values or expectations. Further, experiment results show that moral judgments on human drivers and AVs were similar (Kallioinen et al., 2019). Consequently, many researchers emphasize the importance of including public morality and preference in AV ethics (Awad et al., 2018b; De Freitas et al., 2020a; Savulescu et al., 2019; De Freitas et al., 2020a; De Freitas et al., 2020a). It is important to note that the focus of this study is limited to understanding acceptable AV moral behaviors for the public, which has been underexplored. Thus, technical approaches to implement the system are beyond the scope of this research.

The study that observed lay drivers’ moral reasoning showed that moral emotions are an important part of moral judgment during the AV moral dilemma (Rhim et al., 2020). Accordingly, a comprehensive ethical decision-making framework that explains both intuitive and rational aspects of AV ethical behaviors that answers the following research questions is required: What factors affect ethical behavioral intentions in the AV moral dilemma? How do these variables shape ethical behavioral intentions? To answer these questions, this study aims to synthesize a framework that uses the dual-process theory of moral reasoning Greene et al. (2001) to explain and predict pluralistic moral reasoning in the AV moral dilemma.

This study attempts to provide descriptive ethics to enhance understanding of the broad ethical phenomena of the AV moral dilemma by providing a conceptual framework with propositions. The assumption that acceptable or understandable AV behaviors can be learned from the existing data should be avoided De Freitas et al. (2020b), because there are not enough AV crash cases and the discussion of acceptable AV moral behaviors is not finalized. As a result, it is neither possible nor realistic to provide normative guidance that lists how AVs “ought to” behave. Moreover, the established normative AV ethics may not be adequate as AV technology would advance in unexpected ways, or user values may evolve while using the technology. Also, once AVs are embedded in daily lives, it would be difficult to modify AV decisions and policies (Vrščaj et al., 2020). Thus, making normative ethical rules should be done with caution (Dubljević, 2020). In summary, the purpose of this research is to propose a comprehensive conceptual framework called the “Integrative ethical decision-making framework for the AV moral dilemma,” which theorizes that individual characteristics and perceived seriousness of the AV moral dilemma are antecedents of intuitive and rational moral judgments. The contributions of this study are as follows. First, this study provides explanations for the dual-process theory of ethical decision-making during the AV moral dilemma by including both the cognitive and affective mechanisms as integral aspects of AV ethics. Second, this study emphasizes the importance of how the issue is framed instead of focusing only on the impact of a specific moral doctrine to explain flexible and versatile moral judgment during the AV moral dilemma. Last, this study provides a holistic view of how ethical decision-making occurs in the unknown and vague context of the AV moral dilemma, by providing definitions of moderating variables with explanations and propositions.

## Background

### Review of Theoretical Ethical Decision-Making Approaches

Extending human morality literature into artificial agents may facilitate the articulation of computational models (Wallach, 2010; Malle, 2016; Cervantes et al., 2020). Therefore, having a comprehensive understanding of the existing moral judgment theories is crucial to building realistic and accountable AV ethical behaviors. The definition of ethical decision-making is “a process by which individuals use their moral base to determine whether a certain issue is right or wrong” (Carlson et al., 2009, p. 536). Researchers from multiple disciplines have proposed a number of theoretical and conceptual frameworks to explain, predict, and learn about human moral reasoning. Although moral judgment models are not specifically devised to explain AV ethics, some of the representative models have evolved over several decades to provide comprehensiveness to explain complex moral dilemma scenarios, which offers general applicability to other fields (S. D. Hunt and Vitell, 2006). Therefore, understanding the human moral reasoning will provide possible explanations of how moral judgment will occur in the AV moral dilemma.

Traditional moral reasoning approaches are based on rationalist approaches, which posit that people make conscious and intentional ethical decisions (Vitell, 2003). Recently, social psychologists began to focus on the nonrational or intuitionist approaches in moral reasoning by emphasizing the importance of intuition and emotions in moral reasoning (Haidt, 2001; Sonenshein, 2007; Dubljević et al., 2018). Therefore, this study attempts to gain significant insights from a theoretical investigation of the dual-process theory Haidt (2001), Kahneman (2003), Evans (2008), Zollo (2020) by understanding both the rationalist and intuitive approaches to explain socially acceptable AV ethical behaviors.

### The Rationalist Approach

Rest’s model has inspired rationalist ethical decision-making frameworks in the literature across many disciplines (Beu and Buckley, 2001; Dubinsky and Loken, 1989; Ferrell and Gresham, 1985; S. D. Hunt and Vitell, 1986; Jones, 1991; Trevino, 1986). The rationalist approach of ethical decision-making can be summarized as representing a cognitive perspective of an individual, which is rational, controlled, deliberate, intentional, and conscious. The most widely acknowledged ethical decision-making framework is the four-component model by Rest (1986), which is the foundation of most models (Groves et al., 2008). Rest’s model, as well as the majority of ethical decision-making frameworks, begins when a person recognizes that there is an ethical issue, which is called the *Recognize Moral Issue* phase. If an ethical issue has been recognized, an individual’s reasoning moves on to the next step of *Make Moral Judgment*, which is an individual’s cognitive process to “judge which course of action is morally right” (Trevino, 1992, p.445), then the third step called *Establish Moral Intent* follows. This is a cognitive moral development phase that occurs after making a moral judgment Kohlberg (1969), Rest (1986), in which people prioritize their moral values to determine appropriate ethical behaviors. The last step is *Engage in Moral Behavior*, in which an individual makes actions based on his or her moral intentions. These four phases describe the moral reasoning of individuals to be “intentionally rationalize, re-evaluate, and justify, moral standards, rules of conduct, and moral life” (Zollo et al., 2018, *p*.694).

**FIGURE 1:**
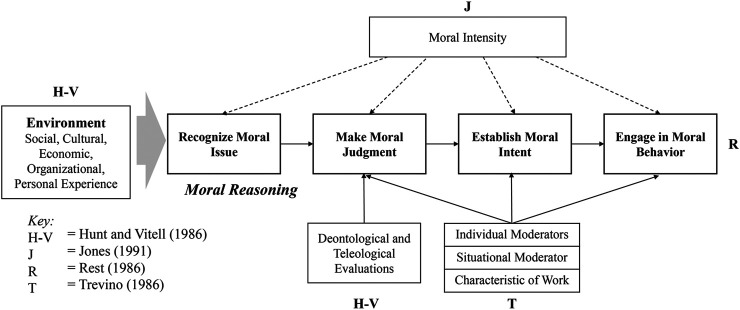
Rationalist approach of ethical decision-making.

The following are the examples of rationalist ethical decision-making frameworks from the multidisciplinary literature that are based on Rest’s (1986) Model. The contingency framework by Ferrell and Gresham (1985) describes that an individual’s moral reasoning begins when he or she faces an ethical salient context. This model synthesizes multiple variables to explain whether an individual’s behavior is ethical or unethical. An individual’s moral reasoning is influenced by the following factors: individual (i.e., knowledge, values, attitudes, intentions), significant others (i.e., differential association, role set configuration), and opportunity (i.e., professional codes, corporate policy, rewards/punishment). This model also includes social and cultural environmental factors that shape an individual’s ethical intentions. The Person-Situation Interactionist model by Trevino (1986) implements the stage of Kohlberg’s cognitive moral development (Kohlberg, 1969) as an integral predictor of ethical behavior. Moral judgment in Trevino’s model is moderated by both an individual moderator (i.e., ego strength, field of dependence, and locus of control) and a situational moderator (i.e., immediate job context, organizational culture, characteristic of the work). The general theory of marketing ethics of Hunt and Vitell (1986) was developed to reduce the ethics gap between the marketers and the society by providing a general ethical decision-making theory with a visible process model (S. D. Hunt and Vitell, 2006). This model is similar to the Contingency framework by Ferrell and Gresham, (1985) as both acknowledge the impact of external factors (i.e., cultural, industry, and organizational environment) and individual factors in moral judgment. However, the Hunt and Vitell (1986) model explains that individuals use specific moral doctrines (deontological or teleological) to evaluate and determine ethical consequences during perceived ethical problem stages. That is, this model puts emphasis on the micro aspects of an individual’s cognitive decision-making process. Jones’ (1991) issue-contingent model includes the four moral reasoning phases like other models and proposes that environmental factors and individual factors positively impact the ethical decision-making phases. On top of this, Jones (1991) emphasizes the moral intensity of a particular context (see Table 1) for further definitions and application for AV ethics). A comprehensive rationalist ethical decision-making framework is illustrated in (Figure 1). While these models provide variables and their relations that explain how individuals perform moral reasoning, they focus on a rationalist approach. Thus, the rationalist approach does not consider the role of emotions or intuitions, which are integral components of moral value codes derived in the AV moral dilemma (Rhim et al., 2020).

**TABLE 1:** Characteristics of AV moral dilemma vignette (source: Rhim et al., 2020, p. 44).

Vignette description	Crash option and result	Moral conflict description
The participant is a driver (V1) who is driving a truck at a two-lane road in a rural area. There are three small passenger cars (V2, V3, and V4) and a truck (V5) on the road. Suddenly, (V2) is changing lane, and a head-on collision with (V1) is expected. There are two crash options with known consequences, and the participant has to choose an option from the perspective of the driver (V1)	The truck (V1) brakes and turns right. This will lead to a collision between the truck (V1) and a small passenger car (V2). As a result, the driver of (V1) will get a minor injury, while the driver of (V2) has died	Whether to make a self-protecting decision that results in the death of the negligent driver
	The truck (V1) turns right. This will lead (V1) to deviate off the road and collide into a utility pole. As a result, the driver of (V1) will be seriously injured while all the other drivers are intact	Whether to make a utilitarian decision to save a life on behalf of sacrificing oneself when one is not at fault

### The Intuitionist Approach

Researchers have realized that the dominant rational perspective fails to convey the full spectrum of the ethical decision-making processes (Chatzidakis et al., 2018; Cherry and Caldwell, 2013; Yacout and Vitell, 2018). The premise that moral agents are rational decision makers disregards the impact of nonrational or intuitive elements such as emotions and intuition in moral judgment (Sonenshein, 2007; Ruedy et al., 2013; Chowdhury, 2017). Consequently, researchers began to acknowledge the significance of intuitive approaches in ethical decision-making, which include consideration of moral values, emotions, and intuitions (Cherry and Caldwell, 2013; Dedeke, 2015; Haidt, 2001; Haidt and Joseph, 2004; Zollo, forthcoming; Zollo et al., 2017). Haidt (2001) defines moral intuition as “the sudden appearance in consciousness of a moral judgment, including an affective valence (good-bad, like-dislike), without any conscious awareness of having gone through steps of searching, weight evidence, or inferring a conclusion” (p.818).

The dual-process theory of human cognition Kahneman (2003), Evans (2008) explains that moral intuition is an automatic response antecedent to rational moral reasoning (Haidt, 2001; Sonenshein, 2007). The social intuitionist model Haidt (2001), among the most well-known intuitionist models, adopts the dual-process theory and accentuates the role of moral intuition as the initial stage in moral reasoning (Greene et al., 2001; Cushman et al., 2006; Zollo et al., 2017). The theory explains that when the decision maker experiences a morally salient context, he or she makes moral judgments based on intuitions, followed by the post hoc rationalization of moral reasoning. In summary, Haidt (2001) explains that emotive intuition occurs quickly and effortlessly, whereas cognitive reasoning occurs slowly and requires efforts.

Another well-known dual process theory includes the notion of *System 1* and *System 2* (Evans, 2008; Kahneman, 2003). Under this theory, human cognition comprises two information processing systems, which also apply to the ethical decision-making process (Zollo, 2020). *System 1* is the intuitive, effortless, fast, reflexive, and nonconscious cognitive process (Dane and Pratt, 2007). “Intuiting” can be interpreted as *System 1*, which allows a moral agent to make a holistic and intuitive moral judgment during dynamic and uncertain situations (Dane and Pratt, 2007). The next phase, *System 2*, is the controlled, reflective, and analytical cognitive moral reasoning process (Zollo et al., 2017). Basic emotions that arise effortlessly and unconsciously are part of *System 1* (i.e., fear, surprise, and sadness), whereas *System 2* includes more complex emotions that are derived from deliberate, and rational cognition (i.e., disgust, anguish, relief, and embarrassment) (Metcalfe and Mischel, 1999; Zollo et al., 2017). Adopted from Zollo (2020), Figure 2 shows the dual process of ethical decision-making, which includes both moral intuition (System 1) and cognitive moral reasoning (System 2). A more recent study in neuroethics introduced the Agent–Deed–Consequence (ADC) model of moral judgment, which follows an integrative approach to explain moral intuitions (Dubljević and Racine, 2014). More specifically, the ADC model posits that “moral judgment relies on positive and negative evaluations of three different components of moral intuitions: the character of a person; their actions; and the consequences brought about by the given situation” (Dubljević et al., 2018, p.2). The ADC model is simple yet effective in verifying and explaining whether a behavior is ethical or not. Overall, the moral intuitionists Cushman et al.(2006), Greene et al. (2001), Haidt (2001), Sonenshein (2007), Tenbrunsel and Smith‐Crowe (2008), Zollo (2020), Zollo et al. (2017) agree that “moral judgments arise as intuitions generated by automatic cognitive processes, and that the primary role of conscious reasoning is not to generate moral judgments, but to provide a post hoc basis for a moral justification” (Cushman et al., 2006, *p*. 1982). Recent literature on the ethics indicates that considering both the rationalist and intuitive approaches provides a complete understanding of human moral reasoning. Moreover, as AV accidents impose hazards for both individual AV user and the traffic users around the AV user, consideration of intuitive moral judgment along with rational judgment to consider overall impact for the society is important. Consequently, the AV ethics should be in line with the dual-process theory and consider both the rational and intuitive moral judgment phases to discuss socially acceptable AV morality.

**FIGURE 2:**
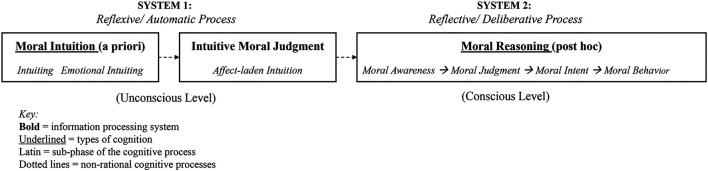
Dual-process model of ethical decision-making (Source: Zollo, 2020, p.7).

### Linking Ethical Decision-Making and AV Ethics

The various ethical decision-making frameworks listed in the previous sections are effective at providing explanations for how moral reasoning variables shape an individual’s ethical intentions. Many researchers agree with the necessity of formulating AV ethics frameworks for varying reasons. First, providing a formal specification of AV moral behaviors will aid other traffic users (e.g., cyclists and pedestrians) to have a better understanding of AVs (Dogan et al., 2016; Mermet and Simon, 2016). Second, an appropriate AV ethics framework helps decision-makers advance responsible AVs that align with societal values, Stilgoe et al. (2013), which can mitigate conflicts between potential harms when adopting AVs (Leikas et al., 2019; Vrščaj et al., 2020). Third, a comprehensive AV ethics model will facilitate translating vague real-world moral theories into machine operationalizable codes by reducing abstractness (Bonnemains et al., 2018).

Several AV ethics frameworks were developed in an attempt to fulfill these goals. Karnouskos (2020b) has utilized the utilitarian principle to explain the acceptance of AVs. Although this model is based on empirical findings, it relies only on a single ethical approach, which can lead to biased decisions. To overcome this limitation, Karnouskos (2020a) has verified that multiple moral frameworks (e.g., utilitarianism, deontology, relativism, absolutism, and pluralism) impact the acceptance of AVs. However, these models do not take into consideration situational or individual factors that impact ethical decision-making. While Smith (2019) has concluded that personality (Honest-Humility vs. Conscientiousness) and ethics positions (Idealism vs. Relativism) impact moral judgment during AV accidents, the model has a gap in explaining the procedural relationships among the variables. The “Generalized Framework for moral dilemmas Involving AV” categorizes layers of factors (cast of characters, vehicle assemblage, and perspective) and suggests four research agendas (Novak, 2020). However, Novak’s model does not have clear definitions of concepts and their interrelations that explain the moral judgment process. While the aforementioned AV frameworks aim to provide accountable and transparent AV ethics, these models do not consider intuitive moral reasoning phases. Furthermore, these models cannot explain the pluralistic ethical decision-making of AV ethics required in complex and dynamic real-world crash contexts. To provide holistic explanations of ethical decision-making during the AV moral dilemma, this study aims to develop a comprehensive AV ethics framework by integrating both the intuitionist and rationalist moral reasoning approaches and understanding how individual and situational characteristics affect ethical decision-making phases.

## Methodology

**FIGURE 3:**
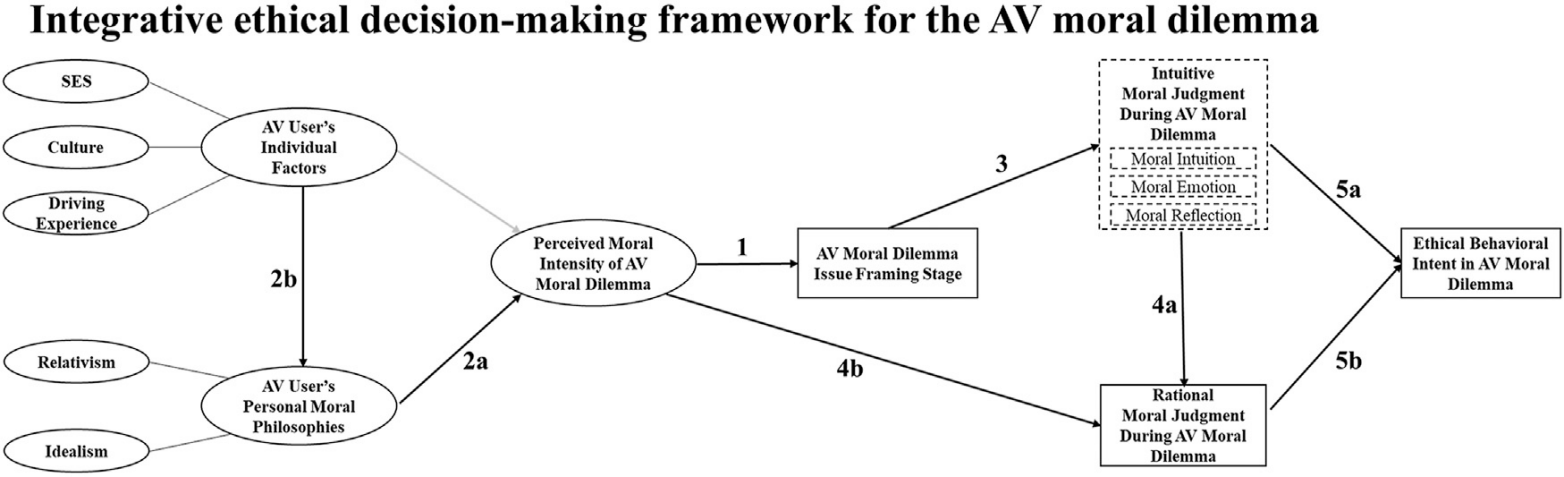
Integrative ethical decision-making framework for the AV moral dilemma.

The theorization of explainable pluralistic AV ethical decision-making is based on the conceptual analysis method to “generate, identify, and trace a phenomenon’s major concepts, which together constitute its theoretical framework” by linking together knowledge from multidisciplinary backgrounds (Jabareen, 2009, p.53). A conceptual framework is the end result of this method, which provides a broader understanding of the phenomenon of interest by providing explanations of possible relationships between concepts (Imenda, 2014; Liehr and Smith, 1999). Moreover, a conceptual framework lays a foundation for research questions and hypotheses for further investigation (McGaghie et al., 2001). This study follows the research stages of Eizenberg and Jabareen (2017), Jabareen (2009) to develop “Integrative ethical decision-making framework for the AV moral dilemma” depicted in Figure 3. First, multidisciplinary literature was reviewed in search of relevant concepts for the AV moral dilemma (e.g., ethics, psychology, sociology, traffic, law, machine ethics, and AI ethics). Second, the reviewed literature was categorized. As the moral reasoning process occurs when an individual perceives a morally salient context, the literature was classified to identify three initial categories: moral reasoning phases, individual factors, and situational factors impacting ethical decisions during the AV moral dilemma. Third, specific concepts were identified. For the moral reasoning category, four interdependent ethical decision-making stages were defined. Both intuitive and rational moral judgment stages were included to describe the dual-process and pluralistic nature of human moral reasoning. Concepts and propositions for both intuitive and rational moral judgment stages include moral value codes that were derived from the AV moral dilemma ethical decision-making process (Rhim et al., 2020). For the individual factors categories, concepts that describe the characteristics and ethical stance of an individual were identified. For situational factors impacting the moral reasoning phases, a variable called perceived moral intensity (PMI) was selected, which evaluates multiple aspects of the AV moral dilemma. PMI includes the perception of risk and uncertainty, important features to consider during AV accidents Kruegel and Uhl (2020); therefore, these two latter concepts were not included separately. Last, the selected concepts were synthesized to provide a comprehensive explanation of how ethical behavioral intentions are shaped during AV moral dilemmas. Further descriptions of the “Integrative ethical decision-making framework for the AV moral dilemma” will be provided in the next section.

## The Proposed Model: Integrated AV Ethical Decision-Making Framework

No matter how complicated AVs are, they are products that can be represented as an extension of their users, owners, or occupants, as the driving task of AV is becoming a comanaged task with humans (Smith, 2019; Bellet et al., 2011). Therefore, the authors posit that AV users will better understand, accept, and trust AVs that make moral judgments similar to oneself. To explain the AV ethical decision-making process during the AV moral dilemma, we have reformulated an integrative ethical decision-making model that includes both the rationalist and intuitive approaches based on previous models (Singhapakdi et al., 1999; Haidt, 2001; Dedeke, 2015; Schwartz, 2016; Zollo, 2020). Aligned with Haidt (2001), Zollo (2020), our framework is descriptive, which describes how people are likely to make ethical intentions during the AV moral dilemma. This study defines the AV moral dilemma as an unavoidable crash situation in which an AV user must reflect upon competing moral standards and determine the appropriate moral behavior of an AV. Moreover, this model posits that moral judgment will vary depending on the individual (e.g., different individuals may perceive varied levels of moral saliency when faced with the same AV moral dilemmas) and situational characteristics (e.g., the same individual may behave differently depending on the characteristic of AV moral dilemma one is facing). The ADC model (Dubljević and Racine, 2014) is one of the most up-to-date and effective models to explain the flexible moral judgment of AVs and overcome the limitation of relying only on utilitarian AV ethics (Dubljević, 2020). The framework developed in this study is complementary to the ADC model. As the components of the ADC model indicate, the model assesses ethical consequences based on deeds of agents. While the Theory of Planned Behavior (Ajzen, 1991) links intention and behavior, studies in ethics demonstrate that how an individual intends to act may not necessarily lead to actual ethical behaviors during the moral dilemma (Weber and Gillespie, 1998). Consequently, understanding ethical intentions will provide further insights into why a certain ethical behavior or deed occurs. The “Integrated AV ethical decision-making framework” (Figure 3) in this study describes how ethical behavioral intentions are shaped with specific variables that need to be considered during the AV moral dilemma.

The “Integrated AV ethical decision-making framework” consists of two major components:1) the ethical decision-making process (intuitive and rational) and 2) variables (or factors) that influence the ethical decision-making process. The ethical decision-making process is composed of four stages: AV moral dilemma issue framing, intuitive moral reasoning, rational moral reasoning, and ethical behavioral intention making stages which reflect Rest’s (1986) basic process framework. The ethical decision-making variables include 1) individual factors and 2) personal moral philosophy (PMP), and 3) perceived moral intensity (PMI). The model consists of 9 links, which are shown in arrows in Figure 3. The solid boxes represent mental state, and the dotted boxes represent mental processes. The current model assumes that accountable ethical behavior of an AV is contingent on the particular AV moral dilemma context that an individual faces. In summary, the “Integrated AV ethical decision-making framework” explains pluralistic nature of AV ethics by investigating how context-specific ethical intentions are shaped during the AV moral dilemmas.

### AV Moral Dilemma Issue Framing Stage

**FIGURE 4:**
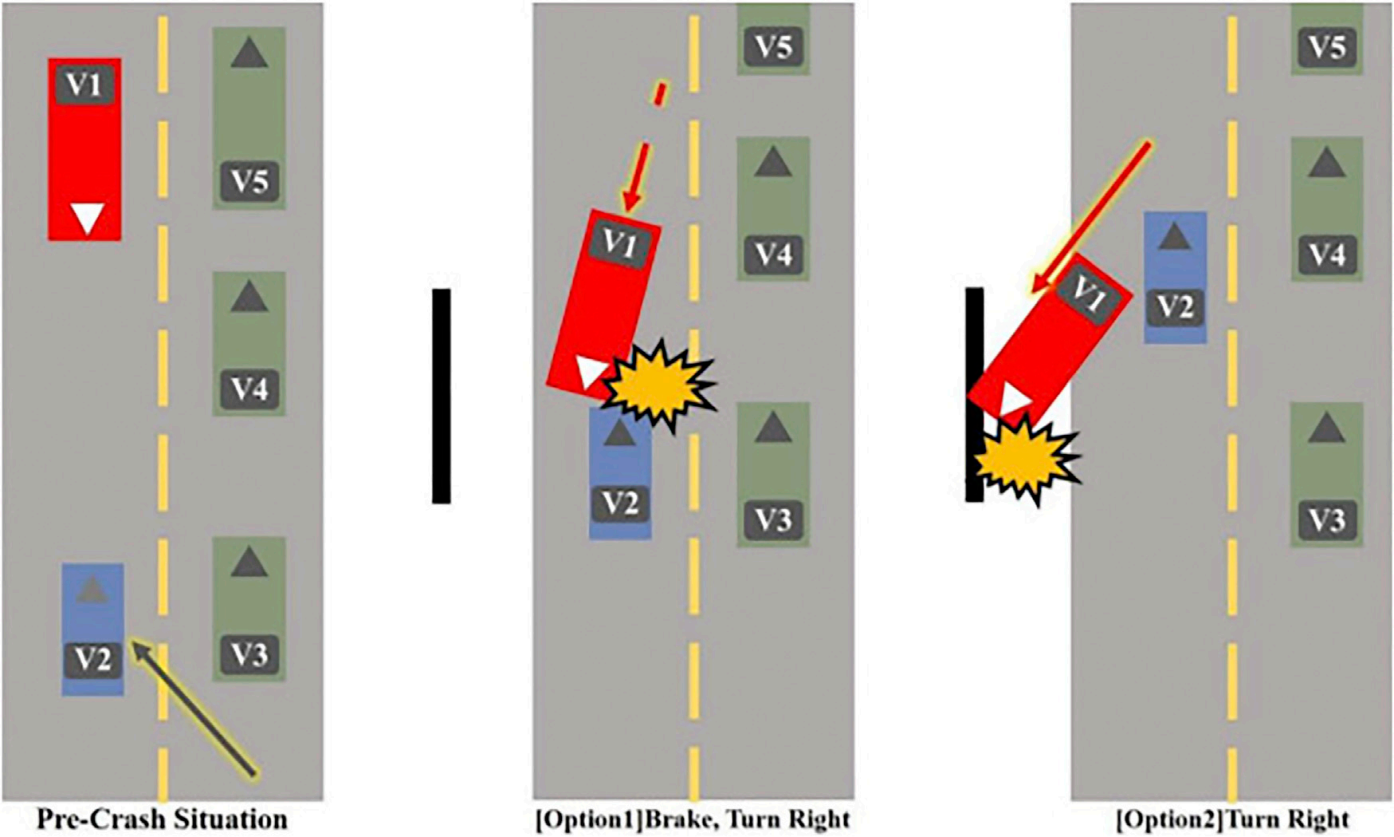
AV moral dilemma: a rural two-lane road (source: Rhim et al., 2020, p. 45).

It is widely accepted that moral judgment is based on how an individual perceives the moral issue rather than the actual characteristics of the issues (Jones, 1991; Robin et al., 1996; A. E. Tenbrunsel and Messick, 1999; Trevino, 1986). That is, the situational context impacts an individual’s unique moral frame, which is a key component in the ethical decision-making process. It is highly likely that each AV crash's characteristics will be unique (e.g., number of passengers in the car, severity of the injury, damage done to one’s vehicle, liability, relationship to the injured victims), and understanding how an individual frames the specific AV moral dilemma is important. According to Rhim et al. (2020), how participants framed the moral issue impacted their AV moral dilemma decisions. For instance, in the AV moral dilemma vignette three (see Table 2; Figure 4) that involved the conflict between making a self-protecting decision or following a utilitarian doctrine to minimize the overall harm, the individual’s moral value code (e.g., Harm Mitigation vs. Self-Preservation) determined their decisions. Furthermore, locus of control is known to impact the moral issue framing stage (Forte, 2005; Dedeke, 2015). In the case of AV moral dilemma, when the locus of control was perceived as internal (making decisions in the first-person perspective), participants’ ethical decisions varied (e.g., moral values: kin-preservation, pedestrian-preservation, physical harm avoidance, and responsibility distribution) (Rhim et al., 2020).

**TABLE 2:** Definition of moral intensity factors.

Factor	Definition [source: Jones (1991)]	Example in unavoidable AV crashes
Magnitude of consequences	“Sum of harms (or benefits) done to victims (or beneficiaries) of the moral act in question” (p. 374)	The AV’s decision that causes the death of a person is more consequential than the one that causes a minor injury
Social consensus	“The degree of social agreement that a proposed act is evil (or good)” (p. 375)	The AV’s decision to protect law-abiding pedestrians has a greater social consensus than a decision to protect the AV driver who has caused the accident
Probability of effect	“A joint function of the probability that the act in question will actually take place and the act in question will actually cause the harm (benefit) predicted” (p. 375)	The AV’s decision that has the 10% probability of causing a serious injury to one passenger has a lower probability of effect than the decision that causes minor injury to all passengers with 100% probability
Temporal immediacy	“The length of time between the present and the onset of consequences of the moral act in question (shorter length of time implies greater immediacy)” (p. 376)	AV that causes harm to 1% of traffic users within 5 years has higher temporal immediacy than AV that harms 1% of traffic users within 20 years
Proximity	“The feeling of nearness (social, cultural, psychological, or physical) that the moral agent has for victims (beneficiaries) of the evil (beneficial) act in question” (p. 376)	The AV’s decision that harms a passenger who is a family member has a higher proximity effect than when the effect will be experienced by a stranger in a different vehicle
Concentration of effect	“The moral act is an inverse function of the number of people affected by an act of given magnitude” (p. 377)	The AV’s decision that leads to 10 fatalities has a higher concentrated effect than causing fatalities to 5 people

All these findings support the inclusion of the moral issue framing stage from the first-person perspective as the initial stage of ethical decision-making in the AV moral dilemma, in compliance with the Cognitive-Intuitionist Model (Dedeke, 2015). Moral issue framing in this framework posits that individuals organize the characteristics of moral issues based on the perceived seriousness of the AV moral dilemma, which is impacted by individual characteristics. Hence, the following proposition can be made:

**Link 1:** The AV user frames the characteristics of moral issues based on his or her perceived seriousness of the AV moral dilemma.

#### The Consequence of Perceived Moral Intensity of AV Moral Dilemma

Extensive studies show that the characteristics of a moral issue will impact the ethical decision-making process. Characteristics of moral issues can be measured or described by moral intensity, which is defined as “a construct that captures the extent of issues-related moral imperative in a situation” (Jones, 1991, p.372). Moral intensity is composed of six components. See Table 2 for a definition of each component with examples in the AV moral dilemma. This framework focuses on perceived moral intensity (PMI) because it is effective for describing moral perceptions that vary across situations and individuals. For instance, while an individual perceives the moral issue to be of high moral intensity, another individual might perceive the identical issue as being of low moral intensity (Robin et al. (1996) depending on his or her individual characteristics and perceptions of the context (further explained in upcoming sections). Specifically, we posit that an AV moral dilemma that triggers high PMI will cause more extensive moral judgment cycles, while situations that prompt low PMI will lead to less in-depth moral judgment. Furthermore, empirical studies have shown a significant correlation between PMI and moral intents (Dubinsky and Loken, 1989; Ferrell et al., 1998; May and Pauli, 2002; Singhapakdi et al., 1999). Hence, this model expects that PMI will impact the ethical behavioral intent stage. In summary, this model specifies PMI as an integral variable that shapes ethical decision-making in the AV moral dilemma. Specifically, the characteristics of an AV accident will impact how an AV occupant frames the moral issue, which in turn will impact moral judgment and ethical behavioral intentions.

#### The Antecedents of Perceived Moral Intensity

PMI focuses on the exogenous characteristics of the moral situation, excluding traits of the moral decision-maker such as values, knowledge, or moral development (Ferrell and Gresham, 1985; Jones, 1991; Kohlberg, 1969). Therefore, AV users’ innate variables impacting PMP will be explored in the following section.

##### AV User’s Personal Moral Philosophy

Many researchers agree that a decision-maker will utilize ethical guidelines based on their personal moral philosophy (PMP) during ethically salient situations (Ferrell and Gresham, 1985; D. R. Forsyth, 1980; D. R. Forsyth et al., 1988, 2008; A. Singhapakdi et al., 1999; Vitell et al., 1993). Based on the established study results, this model presupposes that AV users will apply ethical guidelines based on their PMP when making ethical evaluations in AV moral dilemmas.

Forsyth (1980) explains that the predictors of an individual’s moral judgments can be described by two nomothetic dimensions of PMP: relativism and idealism. Relativism indicates “the extent to which the individual rejects universal rules” when making ethical decisions. That is, relativists base their moral judgments on skepticism and “generally feel that moral actions depend upon the nature of the situation and individuals involved … more than the ethical principle that was violated” (Forsyth, 1992, p.462). On the other hand, idealists have “concern for the welfare of others … feel that harming others is always avoidable, and they would rather not choose between the lesser of two evils which will lead to negative consequences for other people” (Forsyth, 1992, p.462). Moreover, idealists feel that “desirable consequences can, with the ‘right’ action, always be obtained'' (Forsyth, 1980, p.176). That is, idealists are moral optimists who value altruism.

**FIGURE 5:**
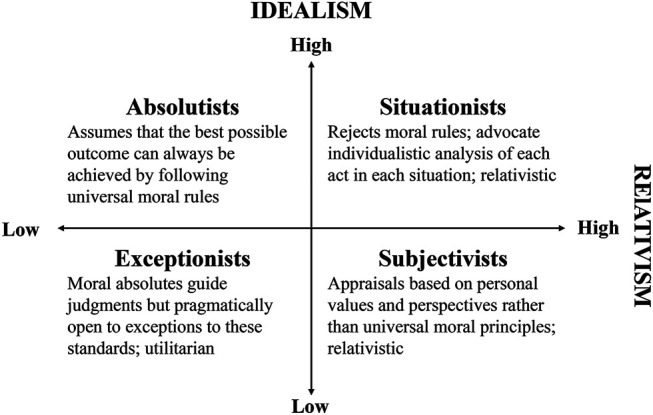
Taxonomy of ethical ideologies (Source: Forsyth, 1980, p. 176).

Forsyth (1980) has classified four dichotomized ethical perspectives based on both dimensions rather than classifying individuals as either relativistic or idealistic, which is called the Ethics Position (see Figure 5). An individual’s Ethics Position (Forsyth, 1980) is formed over a lifetime of experiences and has a strong impact on an individual’s decision-making in a morally salient situation (D. R. Forsyth, 1980; D. R. Forsyth et al., 2008). Research results over the past 2 decades show relatively consistent findings. Idealism had an overall positive relation to moral judgment, whereas relativism had an overall negative relation to ethical decision-making (O’Fallon and Butterfield, 2013). Moreover, PMP has been empirically tested to operate through PMI (D. Forsyth, 1985; D. Forsyth and Pope, 1984; A. Singhapakdi et al., 1999). Based on the previous studies, this model explains that PMP will impact PMI in the AV moral dilemma. Further, this study proposes that AV occupants who score higher in idealism (e.g., who aim to secure the overall welfare of road sharers) and lower in relativism (e.g., who prioritizes protecting oneself more over others) will be more sensitive to ethical issues than their counterparts. Hence, we propose the following propositions:

**Link 2a.** PMP of AV user impacts PMI of AV moral dilemma.

-A more idealistic AV user will have a higher PMI than a less idealistic AV user
-A more relativistic AV user will have a lower PMI than a less relativistic AV user

##### AV User’s Individual Factors as Antecedent of Personal Moral Philosophy

Singhapakdi et al. (1999) emphasized the role of individual characteristics in shaping PMP, which impact PMI and ethical decision-making processes. This study will explore the following individual and cultural factors that are likely to impact PMI in the AV moral dilemma: 1) Socioeconomic status (SES): income and education, 2) culture (or nationality), and 3) driving experience. Moreover, this model posits that individual characteristics impact the moral issue framing stage, which aligns with the model of (Sonenshein, 2007). Thus, the following proposition is developed:

**Link 2b:** Individual factors impact the moral issue framing stage of the AV moral dilemma.

###### Socioeconomic status

####### Income

Despite the scarcity of previous studies, it is essential to explore the impact of SES on the perception of AV ethics for the following reasons. As SES affects AV users’ acceptance of adopting AVs (wealthier people tend to favor and anticipate the adoption of AVs more) (Webb et al., 2019), it is more likely that users with higher SES will adopt AVs first. When a new product or service is deployed, it is natural that feedback from the initial users will be incorporated to modify the product or service. In general, income tends to rise with the advancement of education levels. Relatively few studies explored the impact of income on ethical decision-making. Among a few empirical results, Pratt (1991) found a consistent tendency for higher salary individuals to be more sensitive to unethical actions than those with lower salaries. Moreover, Singhapakdi et al. (1999) found that salary was negatively related to relativism. Similarly, the ethical perceptions of AV users who are higher in SES are highly likely to be referenced more for modifying the ethical behaviors of AVs. Therefore, before an actual system is implemented, it is imperative to explore the PMP of a wide range of SES, which in turn would impact the overall perception of the ethical decision-making process of AVs.

**Link 2b-1:** Income will have an impact on PMP.

-AV users with higher income will be more idealistic than AV users with lower income
-AV users with higher income will be less relativistic than AV users with lower income

####### Education

Studies in ethics have included education (types and number of years) as a variable that impacts ethical decision-making because education is linked to an individual’s cognitive moral development stages (Rest, 1986). Some study results showed significant differences in moral reasoning among individuals with different education levels (Wimalasiri et al., 1996; Latif, 2001; Kracher et al., 2002). For instance, Sparks and Hunt (1998) found that individuals with more domain knowledge were more ethically sensitive than novices. Cole and Smith (1996) found that less educated individuals were more accepting of ethically questionable statements than more educated people. Moreover, people showed a significant difference in recognition of ethical scenarios after receiving education (Wu, 2003).

Singhapakdi et al. (1999, p.23) explain that education shows a noticeable impact on PMP, because “with education may come greater sensitivity to alternative points of view, skepticism regarding moral absolutes, and pessimism that moral dilemmas can always have desirable outcomes.” Moreover, ethical decision-makers in higher education are conventionally at higher moral development levels, thus becoming more aware of people holding varying values or rules that can be relative to one’s norm (Kholberg, 1969). Likewise, AV users with higher education levels are likely situated at higher stages of moral development, which enables consideration of the overall impact of crash consequences. For these reasons, the following propositions are developed:

**Link 2b-2:** The education level of an AV user will have an impact on PMP.

-More educated AV users are less idealistic than less educated AV users
-More educated AV users will be more relativistic than less educated AV users

####### Culture

It is widely accepted that culture influences an individual’s perception of moral dilemmas and the ethical decision-making process (Ferrell and Gresham, 1985; Graham et al., 2013; Hunt and Vitell, 1986; Hunt and Vitell, 2006). Further, it would be neither feasible nor acceptable to develop universally agreed upon AV ethics, as preferred moral decisions vary depending on cultures or countries (Awad et al., 2018b; De Freitas et al., 2020b; Dubljević, 2020). There are various definitions for culture, but one of the most accepted definitions is by Hofstede, which defines culture as “the collective programming of the mind that distinguishes the members of one group or category of people from another” (Hofstede et al., 2005, p.516). As culture includes values, shared beliefs, norms, and ideals Reidenbach and Robin (1991), moral obligations that are socially acceptable in one culture are rejected in other societies, despite the existence of universal moral principles (Mikhail, 2007). Moreover, cross-cultural studies in AV ethics indicated that people from different cultural backgrounds favored different AV moralities (Awad et al., 2018a; Ranasinghe et al., 2020; Rhim et al., 2020).

Forsyth et al. (2008) conducted a meta-analysis to investigate cultural differences by measuring the level of PMP. The review of 139 studies (29 nations, total *n* = 30,230) revealed that idealism and relativism levels vary across cultures in predictable ways and dominant ethics positions existed in each culture: Western culture (subjectivism), Eastern cultures (situationism), and Middle Eastern cultures (absolutism and situationism). The variations of idealism and relativism tend to be uniform with cultural characteristics (e.g., Hofstede and McCrae, 2004; Inglehart and Baker, 2000). Forsyth et al. (2008) explain that regarding idealism, it is predicted that Western cultures adopt less idealistic moral philosophies compared to the Eastern cultures, which can be explained by individualism (a defining characteristic of Western culture). Individualism focuses on the independence of each individual and allows the pursuit of autonomy and free will among groups, whereas collectivism (a defining characteristic of Eastern Culture) prioritizes the goal or well-being of a group before an individual. Thus, Eastern cultures that accentuate a sense of collectivism imply higher idealism than Western cultures. In terms of relativism, it is expected that Eastern cultures will be more relativistic than Western cultures. Eastern cultures tend to be more contextual and relational in comparison with Western cultures (Forsyth et al., 2008). In terms of ethics position, situationism (high idealism and high relativism, see Figure 3) is dominant in Eastern cultures. Situationists posit that an individual should act to secure the most beneficial consequences for all the group members, even if such a consequence is the result of violating moral rules. The situationists’ moral outlook can be described by ethical skepticism or value pluralism, which suggests that the consequences of an action can determine the situation’s moral values (D. R. Forsyth, 1992). On the other hand, Western cultures’ dominant ethics position classification is exceptionist (low idealism and low relativism, see Figure 3), which posits that an individual fundamentally seeks to follow moral rules but is open to pragmatic results. The exceptionist moral outlook highly corresponds to “rule-utilitarianism,” which indicates that “moral principles are useful because they provide a framework for making choices and acting in a way that will tend to produce the best consequences for all concerned” (Forsyth, 1992, p. 463). Cross-cultural studies in AV ethics showed similar patterns. Eastern cultures showed a higher tendency to make context-dependent decisions during AV moral dilemmas (Rhim et al., 2020). On the other hand, the Westerns culture showed a stronger tendency to spare a greater number of people during the AV moral dilemma Awad et al. (2018b), Rhim et al. (2020), which corresponds to the exceptionist moral outlook. In summary, it is expected that cultural background can have a general impact on PMP. Hence, the following propositions are provided:

**Link 2b-3:** The cultural background of an AV user will have an impact on their PMP.

-AV users from Eastern cultures will tend to be more idealistic than AV users from Western cultures
-AV users from Eastern cultures will tend to be more relativistic than AV users from Western cultures
-AV users from Western cultures will generally endorse an exceptionist ethics position (Low idealism, Low relativism)
-AV users from Eastern cultures will generally endorse a situationist ethics position (High idealism, High relativism)

####### Driving Experience

Crashes caused by teen drivers comprise a major part of conventional vehicle collisions. The causes of teen crashes include inexperience in driving and underestimation of perilous driving behaviors (Williams, 2003; Rhodes and Pivik, 2011). Conversely, older drivers are likely to have more experience and have driven longer distances, thus are likely to have experienced situations with a greater variety of ethical problems. As studies that investigate the correlation between ethical decision-making and driving experiences are underexplored, the current study will refer to ethics studies that explored age as a predictor of ethical decision-making, as age and driving experience have a possible association. According to a meta-analysis, more than twenty studies have observed a positive relationship between age and ethical decision-making (O’Fallon and Butterfield, 2013). Study results show that older individuals tend to be more ethically sensitive than younger individuals (Karcher, 1996; Deshpande, 1997; Peterson et al., 2001). Furthermore, older generations made more ethical decisions than younger generations (Hunt and Jennings, 1997; Lund, 2000; Kim and Chun, 2003). In terms of PMP, the literature reveals that a negative association between age and relativism exists, whereas the findings for idealism are inconsistent (D. R. Forsyth, 1980; Ho et al., 1997; Vitell et al., 1991). In summary, it is expected that AV users with more driving experience (both direct and indirect) would be more sensitive to ethical transgressions and provide more suitable moral solutions to novel AV moral dilemma scenarios. Another expectation is that older drivers are more likely to be married and have children of their own than younger drivers, which would impact their commitment to producing outcomes that are more desirable for the overall society (e.g., protect adults who might be parents of children, protect children).

**Link 2b-4:** Driving experience will have an impact on PMP.

-More experienced AV users will be more idealistic than less experienced AV users
-More experienced AV users will be less relativistic than less experienced AV users

### The Intuitive Moral Judgment During AV Moral Dilemma

More researchers emphasize the nonrationalist approach by including intuition and/or emotion in the moral reasoning process (Haidt, 2001; Saltzstein and Kasachkoff, 2004; Cushman et al., 2006; Sonenshein, 2007; Ruedy et al., 2013; Dedeke, 2015; Schwartz, 2016). As unexpected hazards threaten the lives of traffic users during an AV moral dilemma, intuition and/or emotion is expected to be an important factor that impacts the moral judgment stage. Moreover, intuitive moral reasoning is the response to the individual’s framed moral issue. Thus, intuitive moral reasoning mediates the issue framing stage and the rational moral judgment stage. The “Integrated AV ethical decision-making framework” suggests that both intuitive and cognitive reasoning take place, thus supporting the dual-process theory of ethical decision-making (Haidt, 2001; Dane and Pratt, 2007). This section explains the intuitive moral reasoning process. We propose the following proposition.

**Link 3:** The intuitive moral judgment stage mediates the relationship between the AV moral dilemma issue framing stage and the rational moral judgment stage.

#### Moral Intuition

Moral intuiting is a non-conscious cognitive process that occurs quickly and effortlessly Kahneman (2003), Evans (2008) when an individual perceives a morally salient context (Haidt, 2001; Reynolds, 2006; Schwartz, 2016). The dual-process theory explains that intuitive moral reasoning occurs automatically and effortlessly prior to slow and effortful moral reasoning (Greene et al., 2001; Haidt, 2001; Haidt and Joseph, 2004; Greene, 2007; Greene, 2009). However, there is a limitation of this theory. The dual-process theory interprets emotional processes as fast and unconscious, which oversimplifies the moral reasoning process and may neglect the possibility of conscious decision-making (Christensen and Sutton, 2012). Moreover, studies show that people make automatic and unconscious cognitive judgments based on their prior experiences (Greenwald and Farnham, 2000; Bargh et al., 2001; Dedeke, 2015). Consequently, this study does not distinguish intuitive processes as automatic and unconscious and cognitive moral reasoning as slow and conscious but acknowledges that both intuition and cognition can automatically occur during moral reasoning. In line with the previous findings, this framework expects that AV occupants who have not experienced AV accidents can automatically and effortlessly make both intuitive and cognitive responses during the AV moral dilemma because people have intuition and have preliminary moral knowledge in vehicle accidents that can be extended to AV moral dilemma scenarios. In other words, when an AV user faces an AV moral dilemma, a reflexive pattern-matching process may be unconsciously started, and the best prototype that matches the novel context that also matches the user’s values will be more acceptable or understandable for the user.

#### Moral Emotions

Moral emotion has been explicitly included in ethical decision-making (Gaudine and Thorne, 2001; Salvador and Folger, 2009). The following is a categorization of moral emotions that suggest direct relations to ethical decision-making (Eisenberg, 2000; Tangney et al., 2007), which can also be found during the AV moral dilemma: 1) “Prosocial” Moral emotions (e.g., empathy, sympathy, concern, or compassion). Prosocial behaviors such as providing support or help had a link between sympathy (e.g., Carlo et al., 2011), and compassion is activated when the suffering of others is viewed, which leads to altruistic moral actions (Goetz et al., 2010), 2) “Self-Conscious” Moral Emotions (e.g., guilt, shame, embarrassment). Emotions in this category are “evoked by self-reflection and self-evaluation” (Tangney et al., 2007, p. 347). Feeling guilt results from recognizing how the other party has been wronged, and thus leads to empathetic behaviors (de Hooge et al., 2007). 3) “Other-blame” Moral emotions (e.g., contempt, anger, and disgust). People who feel anger tend to attribute blame to others, thus aggregating aggressive behaviors (Dix et al., 1990; Keltner et al., 1993), because anger is often related to justice or fairness (Goldman, 2003). In addition, in a study that explored dual-process reasoning during the AV moral dilemma, moral emotions or related moral value codes in the context of AV ethics that fall into these categories were found (e.g., empathy, conscience, self-sacrifice, children-preservation, kin preservation, passenger preservation, fault liability of self, anger, and fault liability of others) (Rhim et al., 2020). Although AV accidents are a new phenomenon, moral emotions or emotional reactions toward a novel context will allow people to determine what is ethical or not. Therefore, it is crucial to include emotion as a mental process of moral reasoning during the AV moral dilemma. In summary, this framework adopts that individuals will respond to novel AV moral dilemma contexts depending on their emotional responses (Sonenshein, 2007; Dedeke, 2015).

#### Moral Reflection

In conventional crashes, moral reflection would rarely occur since most crash avoidance behaviors are reflexive actions without moral judgment. In contrast, when developing moral behaviors of AVs, the inclusion of the moral reflection stage is possible, which provides the opportunity to reflect upon contexts to minimize conflict that could occur (e.g., consequences vs. fairness). Dedeke (2015) explains that moral reflection focuses on the factual review process, and the role of moral reflection becomes more important when situations involve strong automatic responses, both emotional and cognitive. Thus, the following questions can be asked to reduce bias and minimize immediate reactions based on reflexive judgment. “Do I have all the facts to make my conclusion? Am I interpreting the facts in the correct way? Am I using the correct frame of reference?” (Dedeke, 2015, p.447). In this regard, the moral reflection stage during an AV moral dilemma will promote more accurate processing of information leading to more acceptable decisions for overall society.

Moral reflection occurs after reviewing facts that would occur during a moral dilemma (e.g., what will be the consequences of each decision? Whose liability will it be? What would be the fairest decision?). Reidenbach and Robin (1990) specified dimensions of moral reflection: The relativistic dimension evaluates whether a decision is traditionally acceptable or not and whether it is culturally appropriate or not. Further, the contractualism dimension evaluates whether unspoken promises or unwritten contracts are violated or not. These dimensions are derived from moral philosophies (Reidenbach and Robin, 1990). The relativistic and contractualism dimensions can be referenced in the AV moral reflection stage to induce more ethical and socially acceptable AV decisions. For example, one of the AV moral dilemma scenarios includes “Comply with road traffic laws which results in maximized overall harms” (Rhim et al., 2020, p. 44). An initial automatic intuition would perceive that following traffic rules is ethical. However, if the consequences result in multiple fatalities, the decision may not be ethical nor socially acceptable. As AVs can be preprogrammed, various consequences and reflections should be included in the algorithms. In summary, based on previous studies, this framework emphasizes the role of the moral rationalization process Dedeke (2015), Schwartz (2016), especially after reflexive moral reasoning, because reasoning that occurred quickly may not consider the full spectrum of the problem (Sonenshein, 2007).

### Rational Moral Reasoning During AV Moral Dilemma

This study includes varying factors that impact intuitive and rational moral judgment either directly or potentially in ethical decision-making to explain the dual-process theory in the AV moral dilemma. How rational moral reasoning is shaped and impacts ethical intention will be explained in this section.

#### Rational Moral Judgment

In accordance with Dedeke’s (2015) cognitive-intuitionist model, this framework provides an explanation of pluralistic moral reasoning judgment patterns. First, moral judgment could be mainly based on an AV user’s intuitive reaction toward the framed moral issue. Second, moral judgment could be established mostly on rational judgment, in which intuition is less evoked. Third, a moral judgment could rely on both intuition and rational reasoning. In this case, the automatic reasoning process is the basis for moral reflection and rational reasoning process. In the AV moral dilemma, if one is directly impacted or involved in the AV accident, moral intuition would be more likely to be activated. For instance, if an AV user feels compassion toward pedestrians during an AV accident, he or she will tend to make moral judgments that could preserve pedestrians over other involved traffic users. Or if the decision-maker is a bystander of an AV accident who is not impacted by the accidents, moral emotion would be less significant, and the rational reasoning process will become more dominant. For this reason, intuitive moral reasoning impacts the rational moral judgment process. Moreover, how the decision-maker frames the moral issue impacts the moral reasoning process (Dedeke, 2015). Moral issue frames can explain why people prefer utilitarian AVs, but do not want to buy such AVs. Utilitarian AVs, which intend to save the most lives, seem ethical from the observer’s perspective. However, if the decision is made from the first-person perspective, there is a possibility that the decision-maker can be sacrificed to reduce overall harm. In other words, the moral judgment stage is impacted by how the specific AV moral dilemma is framed by an individual, which is impacted by PMI. Hence, the following propositions are developed:

**Link 4a:** Intuitive moral judgment processes impact rational moral judgment processes.

**Link 4b:** PMI impacts rational moral judgment. For stronger PMI, an AV user will face a more challenging moral reasoning process.

#### Ethical Behavioral Intent in AV Moral Dilemma

An AV user’s contemplation in the moral judgment stage, whether intuitive, rational, or both, leads to the individual’s intention to make either ethical or unethical behaviors during an AV moral dilemma. Researchers agree that emotions impact ethical decision-making. Bagozzi and Pieters (1998) explained that different emotions have discrete goals, thus leading to different behaviors. Moreover, different emotions lead to different moral actions or ethical behavioral intent (EBI) (Blasi, 1999). For instance, the empathy-altruism hypothesis explains that empathy evokes emotions of concern to others who are suffering, which is the driving motivation of altruistic or prosocial behaviors (Batson et al., 1988; Persson and Kajonius, 2016). Similar findings were found in AV moral dilemmas. The dominant moral emotions found for “Moral Altruist” were guilt and empathy. People in this group tend to make decisions that emphasize the safety of overall traffic users, including protecting negligent drivers (Rhim et al., 2020). In the case when cognition is more activated when making EBI, an individual will compare possible actions based on his or her moral principles Bastons (2008) and try to prioritize certain moral values over others to determine moral consequences (Melé, 2005; Craft, 2013). When applied to the AV moral dilemma, an individual’s rational behavior intention would be to minimize overall harm, consider liability, follow road traffic rules, distribute responsibility, or protect a certain party (e.g., cyclists, pedestrians, and passengers in AV). In summary, this study postulates that understanding the impact of both intuition and cognition will provide a more concrete understanding of the connection between moral judgment and moral EBI. Hence, the following propositions are developed:

**Link 5a:** The intuitive moral judgment stage impacts EBI during the AV moral dilemma.

**Link 5b:** The rational moral judgment stage impacts EBI during the AV moral dilemma.

## Discussion

This study illustrates an “Integrative ethical decision-making framework for the AV moral dilemma” to provide an alternative perspective to the conventional trolley problem-based AV ethics. This framework fills in research gaps by explaining pluralistic nature of AV ethical decision-making patterns that reflect the public’s perspectives, which in turn advances social value embedded AV ethics.

The following is the theoretical implication of this study. While many researchers agree with the need for an AV ethics framework to provide explanations of ethical behaviors of AVs, the existing models show only a limited aspect of AV moral reasoning. The “Integrated AV ethical decision-making framework” is one of the first models that provides a comprehensive explanation of the full ethical decision-making process by defining various variables related to the AV moral dilemma. The relationships among the constructs show the step-by-step ethical intention shaping process, which includes both intuitive and cognitive moral reasoning processes. Moreover, the detailed examples and propositions provided in this study overcome the limitation of studies adopting scenario-based methodologies. For instance, understanding the moral issue framing stage may aid in minimizing preconstructed interpretations in the scenarios (e.g., locus of control impacts moral judgment). Therefore, the framework in this study allows consideration of multiple aspects of the AV moral dilemma to discuss realistic AV ethics.

The social contributions of the study are as follows. First, a social value embedded AV ethics framework will provide explainable and transparent AV ethics for prospective users. Singhapakdi et al. (1999) explain that individuals could select ethically questionable decisions simply because they are unfamiliar with the moral issue. Similar trends can be found in AV moral dilemmas because not many people have experienced the novel context of AV involved crashes. Hence, AV instructions based on the framework may help potential users recognize frequently occurring morally salient situations. Moreover, clarification of which ethical decisions of AVs may be more appropriate is likely to enhance recognition of AV crashes with moral saliency and ultimately lead to less unethical AV crash selections. Second, regulators could develop more realistic AV ethical frameworks by considering alternatives to trolley problem-based ethics. Researchers advise that vague AV guidelines should be avoided (De Freitas et al., 2020b). Further, it is widely accepted that regulations are difficult to modify once implemented. Therefore, it is crucial to develop acceptable AVs in the first place. Consequently, establishing realistic and transparent AV ethics would facilitate communication with the public, which will, in turn, increase trust in AV systems. Ultimately, this will prepare the overall society to build socially acceptable AVs.

The following are the technological implications of this study. First, the model offers an alternative perspective to the trolley problem-based AV ethics, which often assumes one moral theory, such as utilitarianism. The propositions provided in this study bring to light that assumptions of ethical behaviors of AVs should be reevaluated (e.g., different cultures will prefer different AV ethical behaviors). Toward addressing this issue, researchers have recently modeled three AV ethical decision-making algorithms (contractarian, utilitarian, and egalitarian) based on a Markov Decision Process (MDP) to react when moral dilemma situation is detected (De Moura et al., 2020). Although the AV decisions from the MDP provide an implementation of pluralistic AV moral behaviors, this model does not consider the intuitive aspect of users. Second, while it might not be feasible to directly program intuitions into AV algorithms, considering moral emotions and the intuiting process that occurs during the AV moral dilemma may enhance prospective users’ acceptance and interpretation of AVs, as well as provide inspirations for engineers. For instance, current AVs are typically programmed with opaque, deep neural networks for fast, low-level processing, along with transparent conditional logic for high-level decision-making (Karpathy, 2020). The level at which to separate these two systems is still an active research topic, including the exploration of completely end-to-end System 1 approaches using reinforcement learning (Kuutii et al., 2020). An analysis of System 1 and System 2 in human ethical decision-making may be a way forward in designing systems that balance effectiveness and explanatory power. Third, human-centered AI (HCAI) provides clear goals to achieve reliable, safe, and trustworthy AI-embedded systems Shneiderman (2020), yet how to achieve these goals is unclear. The variables used in this study such as individual and cultural factors, perceived moral intensity, and possible decision-making patterns can aid engineers in considering machine translatable ethical AV behaviors. For example, in creating AV systems that may be deployed worldwide to different countries, AV developers could integrate tweakable parameters based on situationist vs. exceptionist differences, such as the ability to transgress rules of the road depending on the consequence to the group. As another example, surveys of AV users can be interpreted through the lens of individual factors such as education, age, and their expected moral responses, rather than taken as a whole.

The proposed “Integrative ethical decision-making framework for the AV moral dilemma” is not free of limitations. First, the framework is conceptual and suggests propositions that are not empirically tested. The detailed moral preferences cannot be measured. Future studies could empirically validate the framework Preferences for Precepts Implied in Moral Theories (PPIMT) instrument Dubljević et al. (2018), which “assess respondents’ preference for the precepts implied in the three dominant moral theories” Dubljević (2020), can be used for empirical validation of AV users’ moral judgment tendencies. Measuring PPIMT will provide a more concrete understanding of how the AV moral dilemma context activates users' preference of a specific ethical theory. Second, this model focused mainly on an individual AV user’s moral judgment. However, AVs will be deployed in mixed traffic scenarios where multiple traffic users are involved (e.g., other AVs, conventional cars, pedestrians, passengers, and cyclists) (Nyholm and Smids, 2018; Ranasinghe et al., 2020). The framework or theory can be expanded to describe the interrelationship between multiple traffic users to understand accountable AV moral reasoning in a broader sense. A future study can reference the “Integrated AV ethical decision-making model” when developing social values embedded algorithms and user interfaces. Finally, while this study focused specifically on AV morality, AI-embedded technologies such as social robots will face similar moral conundrums. In the future, this framework may be extended to other related fields to provide a foundational theory to strengthen the field of AI ethics and roboethics.

## Conclusion

This study attempts to fill in research gaps that appear in the existing AV ethics models by providing a comprehensive theoretical framework. It does so by defining key AV moral dilemma-related factors and merging them together into an integrative framework that includes both the intuitive and cognitive moral reasoning processes. More specifically, this study explains how an individual frames the AV moral dilemma, impacted by individual characteristics and PMP, which will in turn be the reference for intuitive and cognitive moral reasoning leading to EBI. The proposed integrated framework can be considered to reflect the “person-situation” interactionist perspective Trevino (1986) as well as the “cognitive-intuitionist” approach (Dedeke, 2015). Consequently, the framework embeds the dual-process theory and provides explanations for moral pluralism of AV ethics that includes the intuitive moral reasoning.

## Data Availability

The original contributions presented in the study are included in the article/Supplementary Material. Further inquiries can be directed to the corresponding author.
